# A qualitative study reporting maternal perceptions of the importance of play for healthy growth and development in the first two years of life

**DOI:** 10.1186/s12887-020-02321-4

**Published:** 2020-09-09

**Authors:** Alessandra Prioreschi, Stephanie Victoria Wrottesley, Wiedaad Slemming, Emmanuel Cohen, Shane Anthony Norris

**Affiliations:** 1 SAMRC/Wits Developmental Pathways for Health Research Unit, Department of Paediatrics and Child Health, Faculty of Health Sciences, University of the Witwatersrand, Johannesburg, South Africa; 2 Division of Community Paediatrics, Department of Paediatrics and Child Health, Faculty of Health Sciences, University of the Witwatersrand, Johannesburg, South Africa; 3 UMR CNRS-MNHN 7206 “Eco-anthropology”, Musée de l’Homme, Paris, France; 4 Global Health Research Institute, School of Human Development and Health & NIHR Southampton Biomedical Research Centre, University of Southampton, Southampton, UK

**Keywords:** Early childhood development, Qualitative, Movement behaviours, Low-to-middle income country

## Abstract

**Background:**

In order for infants and toddlers to meet recommended movement guidelines, their caregivers need to encourage play daily. This study used a qualitative approach to understand how mothers perceive and promote play and physical activity during the first 2 years of life.

**Methods:**

Mothers with children between 0 and 24 months were recruited from the SAMRC/Wits Developmental Pathways for Health Research Unit in Soweto, South Africa. 19 mothers agreed to participate and were grouped into three focus group discussions (FGDs) by age of the child: 0–6-months, 7–14-months, and 15–24-months. Thereafter, 12 mothers (4 from each FGD) were selected for inclusion in in-depth interviews (IDIs). After coding and theme/sub-theme identification had been completed for all IDIs, a process of cross-cutting theme identification and confirmation across FGDs and IDIs was carried out.

**Results:**

The mothers were (mean ± SD) 27 (6) years old. All mothers had attended secondary school, but only nine had matriculated. Only one mother was married (and lived with the child’s father), and the majority (*n* = 15) were unemployed. Most children were male (63%) and were aged 11 (7) months. Four main themes emerged: 1) Physical activity as an indicator for health, 2) Promoting play and development, 3) Gender bias in play, and 4) Screen time.

**Conclusions:**

This study showed that developmental attainment was the most important outcome for mothers, and so focussing intervention content on the promotion of child development through movement is advised. Screen time was freely available to children, and we recommend educating mothers on the movement guidelines, with a particular focus on the detrimental effects of screen time in this age group. Mothers reported many barriers to promoting play, and these are essential to consider when designing interventions in this context, in order to allow for equal opportunities for play to be provided to all children.

## Background

In early childhood, movement behaviours (physical activity, sleep, and sedentary time) have been established as important factors influencing both growth and developmental outcomes [1]. Play is defined as engagement in an activity for enjoyment purposes - and is usually associated with children - the assumption that children will naturally play is common [2]. While play is not meant to be perceived as a task, it is the primary method of engagement in physical activity in the early years. In addition to the health benefits of play, the Nurturing Care Framework describes opportunities for early learning, and responsive caregiving as two of the five key components necessary for optimal early childhood development [3]. Interactive play between caregivers and children can provide an important platform for early learning. In addition, sensitive and caring interactions that are responsive to children’s movements and behaviours can form the foundation for early social interactions and brain development [3]. Therefore, interactive and stimulating play in the first 2 years of life not only ensures that infants and toddlers meet movement guidelines for healthy growth and development, but also ensures that they are receiving the nurturing care they need for optimal emotional and cognitive development.

Guidelines for physical activity in the early years recommend that infants should accumulate sufficient daily tummy time and floor based play, and should not be restrained for extended periods of time [1, 4]. For toddlers, recommendations further advise 180 min of active play per day; and for both age groups no screen time is recommended [1, 4]. However, in the first 2 years of life, infants and toddlers do not have much autonomy over their behaviours, and are reliant on their caregivers providing opportunities for play [3]. Thus, in order for infants and toddlers to meet these guidelines, their caregivers need to understand the importance of play, as well as to encourage play daily [5, 6]. Therefore, parents perceptions of play and physical activity are crucial to understand, when trying to examine or change these early childhood behaviours.

In higher income settings including the USA, Europe, Canada and Australia, factors that have been related to play include access to outdoor space, encouragement of play by caregivers, household socioeconomic status [5], access to indoor play areas, caregiver self-efficacy [6], the physical environment, access to play material, and safety [7]. Evidence is limited in Africa regarding play behaviours and caregiver perceptions. In South Africa, evidence shows that opportunities provided in the home for early learning are scarce, and poverty is likely to be a significant barrier to providing opportunities for development [8]. Furthermore, in lower income settings the social and family set up (including high household density, non-nuclear families with shared caregiving, socioeconomic status and education) [8], and the structural environment (lack of safe space) makes play difficult to incorporate into daily life [2]. Thus, the aim of this study was to use a qualitative approach to understand how mothers perceive play and physical activity during the first 2 years of life, and to understand whether and how they promote this behaviour. In addition, any barriers that may be preventing mothers from allowing their children to achieve optimum activity levels were explored.

## Methods

### Participants

Mothers were purposively recruited from an existing study of mother-baby pairs from the Chris Hani Baragwanath Hospital maternity ward in Soweto, Johannesburg, South Africa in February and March 2018 if their children were between 0 and 24 months. Mothers were included if they consented to participate, lived in the greater Soweto area, were intellectually able to participate in discussions either in English, or in the vernacular language, and were aged 18 or older. Ethical approval was obtained from the University of the Witwatersrand Human Research Ethics Committee and written informed consent was obtained from all participants for both the focus group discussions (FGDs) and the in-depth interviews (IDIs) where applicable, as well as for the recording of their audio. All participants were reimbursed for their transport costs and received refreshments when attending the FGDs and IDIs.

### Data collection

A total of 19 mothers agreed to participate. Participants were grouped into one of three FGDs by age of the child: 0–6-months, 7–14-months, and 15–24-months. Thereafter, 12 mothers (4 from each FGD) were selected for inclusion in the IDIs based on their participation in the FGDs (i.e. those who gave input during the FGDs were selected to participate). All mothers completed a short demographic questionnaire prior to commencement of the FGDs with a trained member of research staff. Both the FGDs and IDIs were initially conducted in English - with flexibility for the participants and facilitator to use vernacular languages – and were audio recorded. Each FGD and IDI was conducted by one of two trained facilitators, while comprehensive field notes were compiled by an observer to supplement the audio files. The FGDs lasted between 60 and 120 min each, and the IDIs lasted between 60 and 90 min each.

**Fig. 1:**
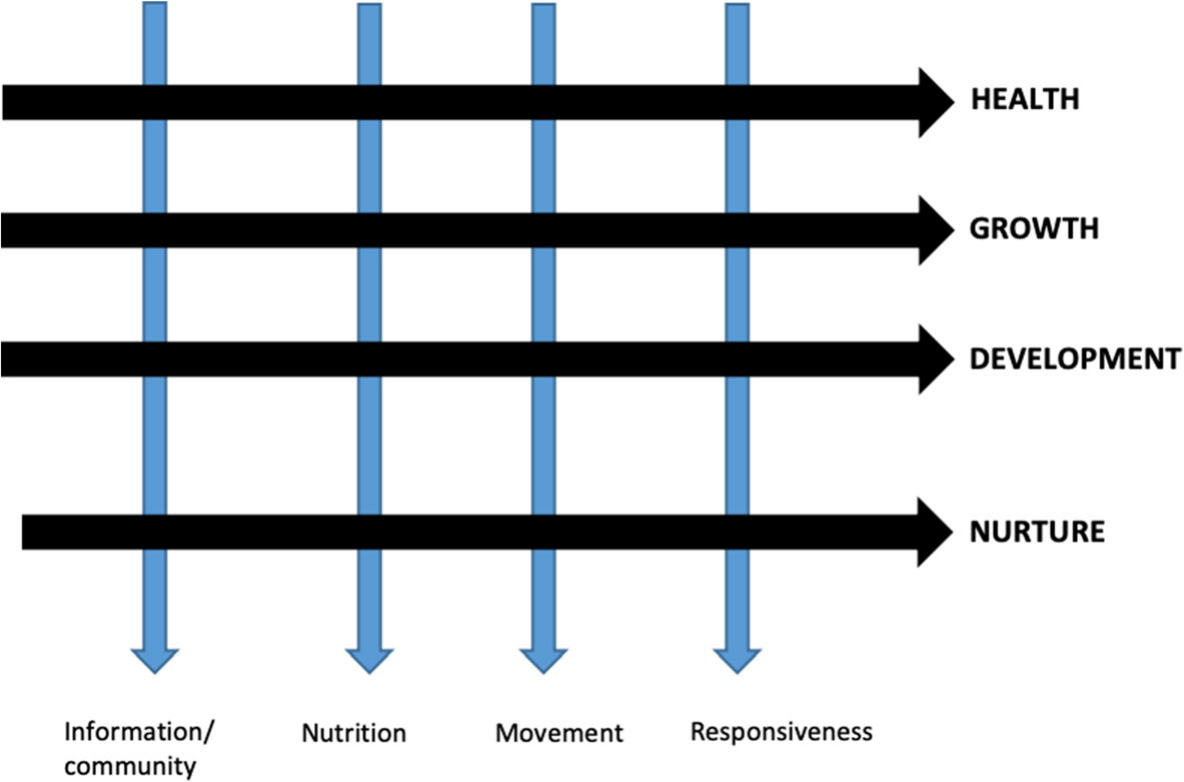
Framework used in the development of the FGD guide, including main topics, and proposed cross-cutting topics to be explored

The FGDs were facilitated using a flexible semi-structured guide (Supplementary Document 1), which was developed according to the framework presented in Fig. 1. This framework was developed by the authors based on four key components essential for early childhood care (health, growth, development, and nurture) [3]; with questions pertaining to four cross cutting topics of interest, that each intersected all four essential care components (information, nutrition, movement and responsiveness) in order to understand how those topics were related to early childhood care. Facilitators encouraged interaction and discussion between participants, as well as brainstorming of topics around their child’s health and wellbeing, movement, and development, allowing for an exploratory discussion. Emerging themes from the FGDs were used to develop and refine the IDI guides (Supplementary Document 2), which were designed to provide a deeper understanding of the topics that emerged during the FGDs, and explore any gaps identified during FGD analyses. However, this interview guide remained semi-structured in order to promote open discussion around the topics and allow for exploration into topics. After completion of both the FGDs and IDIs, the audio files were transcribed verbatim and translated where necessary.

### Data analysis

The three FGD transcripts were divided between three researchers who used a combination of deductive (pre-identified themes based on the research question) and inductive (emerging themes from the transcripts and field notes) approaches to identify and analyse themes. Thereafter, transcripts and coding frameworks were swopped to cross-check interpretation and theme identification across the group. Researchers provided each other with individual reviews of the coding frameworks, thereafter meetings were held to compare, contrast and discuss emerging themes, while incorporating the principles of the immersion-crystallisation method. A fourth researcher then tested reliability and internal validity of the data coded from the FGDs and, where differences were established, these were discussed and resolved as a group. The independent researcher then cross-checked and collated common and unique themes across the three transcripts to develop an overall data codebook for the three FGDs. Following coding, a group meeting was held to complete data analysis and interpretation of themes and the codebook was interrogated and refined until the point that no new themes emerged. This codebook was used as a basis for coding the IDIs. After coding and theme/sub-theme identification had been completed for all IDIs, a process of cross-cutting theme identification and confirmation across FGDs and IDIs was carried out. For the purpose of this study, only the themes and sub-themes related to movement, play and development are reported. In order to illustrate the themes and subthemes that emerged from the FGDs and IDIs, exemplar quotations were excerpted. Finally, data were presented according to the main themes that emerged for this topic.

## Results

The mothers were (mean ± SD) 27 (6) years old. All mothers had attended secondary school, but the total years of schooling ranged from 9 to 12 years (nine had matriculated). Only one mother was married (and lived with the child’s father), and the majority (*n* = 15) were unemployed. Most of the mothers (*n* = 13, 68%) had more than one child. Most children were male (*n* = 12, 63%) and were aged (mean ± SD) 11 (7) months.

Four main themes emerged and the results are presented accordingly: 1) Physical activity as an indicator for health, 2) Promoting play and development, 3) Gender bias in play, and 4) Screen time. Some of these themes were further divided into subthemes and are discussed accordingly. Exemplar quotes are presented in Table 1, as well as in text.

**Table 1:** Exemplar quotes by themes and subthemes

Exemplar Quote	Source	Characteristics
**Physical activity as an indicator for health**		
“You can see from how he plays that he’s fine if he’s not playing you can tell that there’s something wrong or that he’s not feeling okay in his body”	IDI 8	mother of a 13 month old boy
“… when they’re overweight, even when they breathe they don’t breathe properly, when they play with other kids they can’t run… because they’re overweight they get blocked [breathing], some get blocked quickly; so a fat child, an overweight child, no they’re not attractive”	IDI 4	mother of a 6 month old boy
“…he is too much to handle, but he makes me happy, he keeps me busy; when he’s not around… like even now if he were to arrive I’ll be dealing with a lot, but I miss him”	IDI 6	mother of a 8 month old boy
“I think he’s too hyper active, but it’s fine, it’s good. Because he shows me that he’s okay, and I’ll see that there’s something wrong or that he’s sick by him not being active, by not playing”	IDI 11	mother of a 19 month old boy
**Promoting play and development**		
***a) Types of play***		
“Yoh I run after her when I feed her, I have to run after her she doesn’t sit down because it’s time to eat. She eats while walking around so we’ll take 2 h because I have to follow her around so she can eat now and finish now. I have to run after her as if we’re playing”	IDI 5	mother of a 7 month old girl
“Running, playing with toys and singing that they do at crèche we also do at home; we sing, we do numbers, we do 7 days of the week…”	IDI 6	mother of a 8 month old boy
***b) Barriers to play***		
“Ja, only in the house. There’s no space in (my) own yard. Like fence, the razor fence like, (brief silence) and even in the toilet (s)he can get in the toilet, outside toilet it doesn’t have a door”	IDI 2	mother of a 2 month old girl
“Ja because people steal kids these days so we always lock the gate so when someone tries to come in, we can hear it”	IDI 10	mother of an 18 month old boy
“…help that we need is safe spaces, we’re used to our environment but it’s not safe, because there’s… we’re scared of the incidents such as the one I told you about, with the child being kidnapped; and we don’t have parks, there ones that are there are far and it’s not even safe…”	IDI 2	mother of a 2 month old girl
“… the thing that they can help us with are crèches, eish, if we can get proper crèches… because the crèches that our children go to are not alright, there aren’t a lot of toys to play with, like swings and the like for children to play with, and the toys are sparse, so they can help with crèches, and also still on the crèches you find 60 kids to 2 teachers, and the teachers are as young as 22 years and not experienced in looking after children”	IDI 4	mother of a 6 month old boy
“… you want to do certain things for your child, like you want your child to go to crèche, or to be active and play with other kids and you find that you don’t have money, and while the grant money is there, it’s not enough because you have to use that money for food and clothes, and sometimes you find that you don’t get enough support that can help you raise the child, and you end up sitting with the child at home, they don’t speak or do anything and they’re not as active as kids who wander around outside… but to go outside and play with other kids and being around other people generally; I think it helps”	IDI 6	mother of a 8 month old boy
***c) Development in relation to play***		
“Yes, it shows how far the child is still going to go, when you see a child start crawling be inspired that after that my child is going to the next level I have hope that they will walk no matter what”	IDI 12	mother of a 23 month old boy
“My baby was quick to do things so I wouldn’t say there was anything that slowed her down but she was quick to do things ahead of time. On the growth chart it showed that in these months the baby should sit but she was ahead of her time”	IDI 9	mother of a 17 month old boy
“You compare him to the kids his age around the area, you look if he’s body is tinier than the others so he looks small and you look at his mind as well if the others are brighter than him or if he’s fine”	IDI 8	mother of a 13 month old boy
“Besides experience, for example not all of us here have walking rings, or feeding chairs for the child when they’re 6 months we just put the child on our lap and feed them. So there are things that can help a child like a walking ring, but if you don’t have one you have to find other way to help them learn how to stand, maybe let them stand on the couch for a while until they learn to hold onto something and learn to start walking”	FGDs	
“[Interviewer]: Do you read books to her? [Mother]: No! (Laughs) those things only happen on TV, those are white people’s things, I only see that on TV I’ve never done it”	IDI 9	mother of a 17 month old boy
**Gender bias in play**		
“Boys are active, there isn’t anything that they don’t know how to do. Girls are sensitive, when a boy plays with a girl they must know that that is a girl not a boy, he mustn’t be rough with her”	IDI 12	mother of a 23 month old boy
“A girl plays more than a boy and she’s smarter than a boy”	IDI 8	mother of a 13 month old boy
“No, when they play it’s the same, they play the same”	IDI 3	mother of a 4 month old girl
“[Mother]: No I wouldn’t allow him [to buy a doll]. I will say to him “you are a boy”, only gays play with dolls. Yes, my child isn’t gay he’s a boy. [Interviewer]: But he’s still a baby? [Mother]: No he’s going to grow up with it. These kids don’t forget, my child will grow up to be gay”	IDI 12	mother of a 23 month old boy
“When they’re little I think they’re the same like you’ll see a girl playing with a car when a boy comes across a girl’s toy he just takes it so they’re the same when they’re little. I think it depends on the child, it doesn’t matter whether they’re a boy or girl it’s up to the baby how active he/she is”	IDI 9	mother of a 17 month old boy
**Screen time**		
“No, (s)he watches maybe 5 min and then (s)he plays again… like maybe if something is interesting (her) (s)he’ll like focus on TV again…”	IDI 2	mother of a 2 month old girl
“He sits and watch TV, by the sofa, he eats there”	IDI 1	mother of a 1 month old boy
“Yes it’s good because some other cartoons are educational, like Takalani Sesame [South African educational show for children] teaches kids words, and maths and things like that; I think it’s good”	IDI 7	mother of a 12 month old boy
“I wouldn’t let her watch TV from the morning – and she only comes to me when she wants to eat… if there are programmes she watches like Ben 10 airs at a certain time when it’s over, she has to go play because she can’t watch TV the whole day, it makes the kid lazy.”	IDI 5	mother of a 7 month old girl

### Physical activity as an indicator for health

There was a general view that being physically active was a sign that a child was healthy. When mothers were asked how they knew their child was healthy, many reported that they knew if their child “moved a lot”, if they were “busy”, and if they were “lively”. Mothers reported that they would be concerned if their child was too “quiet” or “not playing”. Mothers also mentioned that overweight or underweight were associated with decreased activity.

*“By how lazy [s]he is, if I find that a baby is fat and doesn’t want to do anything I’ll see then that something isn’t right but if [s]he’s active, [s]he’s normal to me” - IDI 5, mother of a 7 month old girl*

Conversely, many mothers also expressed concern about their child’s perceived overactivity. Mothers discussed that, while it was important for their child to be active, it was bad if they were “too active” or “hyper” as they would then battle to “control them”. One mother expressed concern that her child may have ADHD (Attention deficit hyperactivity disorder) because he was “too much”. Mostly, however, mothers reiterated that, even if their child was ‘over active’ and thus making their caregiving more difficult, that they were fine with it because their children made them happy. They also did not express any techniques used to manage perceived over activity.

*“Yes, he’s doing too many things, plus not sleeping early. It’s a bad thing. Maybe he has ADHD I don’t know (laugh). Because aren’t kids who are hyper active, don’t they have ADHD? I have ADHD. I think he has it, he’s too active; I wasn’t active I just had a concentration problem, but he’s too much.” - IDI 1, mother of a 1 month old boy*

### Promoting play and development

This theme was divided into three subthemes: a) Types of play, b) Barriers to play, and c) Development in relation to play.

#### Types of play

Mothers were asked to discuss how they usually played with their children. Most play activities seemed to be naturally occurring, such as playing during feeding time or bath time, talking, singing and dancing. However there was also discussion of more structured play time activities, such as playing with toys, running outside, and using play for teaching moments. Mothers mentioned that they often played while trying to teach their children new words, numbers, days of the week, etc.

#### Barriers to play

Many mothers mentioned safety as a major concern, mainly in relation to outdoor play. These safety concerns were both structural and environmental. Structural concerns related to worry that their child might hurt themselves outside, might fall in the communal outdoor toilet (which raised both safety and hygiene concerns), might land up on the streets (which were reported to be right outside and not always fenced off from the yard), and might get hit by cars - as most mothers reported living on very busy streets. In some cases, mothers reported that they did not have yard space in which their baby could play and therefore the “outdoor area” would be the street.

*“Uhm, the difficulty is the children from where I live like playing in the street, it’s always packed there and cars so it’s not safe for your baby to play outside because space is limited. So she must play in the yard where you can see her because it’s packed there you could be thinking that your baby is playing but someone kidnapped her. These are the issues so she’s not that free to play anywhere, I have to check every now and then if she’s safe.” – IDI 5, mother of a 7 month old girl*

*“He plays but there isn’t enough space… in the house there is big space when we sit and watch TV he plays because there’s big space” - IDI 12, mother of a 23 month boy*

Another major concern reported by most mothers was the fear that their child might be abducted, kidnapped or raped. For this reason, mothers either kept their children inside, or allowed them to play in a locked yard under supervision if they had access to such space. Women also discussed that the structural environment prevented them from promoting play as they would want to. They mentioned that if they had access to safer spaces for children to play, safer and more “hospitable” housing, and safe and experienced day care facilities – they would be better able to promote play sufficiently. Many of the mothers reported financial constraints as a barrier to promoting play. They discussed the fact that, while they wanted to do certain things to promote activity - such as buy toys and pay for creche – they could not afford to. They therefore felt they lacked the support and the agency to achieve this.

*“Sometimes like if you’re at the mall and your baby wants a ball but you don’t have money and a ball keeps your baby active it’s a problem” - IDI 10, mother of a 18 month old boy*

#### Development in relation to play

Mothers were very aware of developmental milestones, and used these to track their child’s development and progress. They also discussed the fact that motor development was related to being active (such as walking or crawling). It appeared that mothers received this information from the clinics and from their Road-to-Health booklets (given to mothers at the child’s birth). Mothers reported a lot of day-to-day comparison between children in the community, whereby mothers became concerned if their child was not developing as fast as others.

*“You tell yourself that maybe your child is slow, and you need someone to talk sense into you, like my child’s father often says to me not to go with what other people are doing, and that my child will teeth it’s just not his time yet, because my child’s father’s grandmother often tried to make me feel bad by pointing out that other kids my child’s age have started teething and yet mine hasn’t.” - FGDs*

Mothers reportedly found ways to promote development, such as those mentioned in the types of play subtheme, and managed to use alternative methods when they could not afford the equipment they felt they needed to promote development. However, there were some misconceptions about the best ways to promote development (i.e.: using a walking ring seemed to be preferable to allowing children to try stand by holding onto tables/couches).

Most mothers expressed that they would like more information on how to promote development and achieve developmental milestones. Mothers told us that they found information online or on the television, or from other family members or friends. Only one mother said that she read to her child, and that she felt that this helped her child to learn. Many of the other mothers said that they did not read, or even own a book.

*“No I don’t read, I don’t even own a book. (Laughs)” - IDI 5, mother of a 7 month old girl*

### Gender bias in play

There was a mixed response to whether mothers thought that boys or girls were naturally more active. Some mothers thought that girls were smarter, and more active than boys, while others stated that girls were naturally more “sensitive” and “whiney”, and should be treated like “eggs” because they are ladies. Some mothers stated that boys were naturally “rough” and “active”, and that playing just came naturally to them. However there were a few mothers who said that boys and girls were the same, or that activity levels depended on the individual child.

*“Ey but boys are rough hey, they’re too active” - IDI 5, mother of a 7 month old girl*

*“Yes, girls are ladies. They know when to stop, and they play properly, boys are rough. I think, I think it’s the treatment. Like girls, girls you treat them like they’re eggs or whatever and then boys, we just allow boys to do anything…” - IDI 1, mother of a 1 month old boy*

Interestingly, a few of the mothers spoke about gender bias in relation to types of play and toys used, and their stereotypical gender assignment. These mothers stated that boys specifically should not play with dolls because then they would become “gay”, and that both them and their elders would insist that this should not be allowed to happen. However, other mothers said that it did not matter what toys or games their children played, especially if they were girls, and that they should be allowed to play however they wanted to.

*“I can’t give my boy child a doll (giggle)*

* [Interviewer] If he chooses the doll himself?*

* No, but he must know about playing with balls and cars; not dolls. I’ll tell him that, that’s for girls.” - IDI 11, mother of a 1 month old boy*

*Yes because girls play with dolls. They don’t want boys to play with dolls*

*[Interviewer] Who?*

*The elders. Because they say he’ll be gay. Yes a boy mustn’t play with a doll it’s worse now because there are a lot of gay people these days*

*[Interviewer] And now is that a concern for you?*

*No I don’t care what he plays with. Mm if he’s gay he’d be gay even if he doesn’t play with a doll” - IDI 8*

*“Yes there should be some for boys and others for girls because at the end of the day if a boy plays with a girl’s toy it’s a problem. He’ll be gay. No because a certain toy was made for a boy (Laughs) for us black people it’s a problem.*

*[Interviewer] What if you’re in the shop and he just picks up a doll?*

*I’d take it from him and put it aside*

*[Interviewer] If he cries for it?*

*Even if he cries for it, I’d never buy it” - IDI 10*

*“Ah it depends if she wants to participate in soccer with boys and she can handle it, she must go play. What if she finds netball boring? She must go play” - IDI 5, mother of a 7 month old girl*

### Screen time

Mothers were asked about their perceptions of screen time for children, and responses were varied. All mothers reported that their children watched some TV, but the amounts ranged from very little: “maybe 5 minutes”, to a lot “maybe about 3 hours”. Some mothers stated that their children preferred to play than to watch TV, or that they got bored, while other children reportedly enjoyed watching TV. Mothers said that their children liked watching adverts or cartoons, while a few said that their children only liked the music on TV. It appeared as if, in most cases, the TV was constantly on in the background and children either specifically sat and watched, or just played or fed in front of, or around the TV, and only engaged with the screen at certain times. The comments gave the impression that most mothers believed that their children had autonomy over their TV time.

*“Yes, he watches TV, like when we’re sitting in the dining room, he’ll watch TV, but he likes adverts a lot of the time, cause even when he’s in the bedroom and they’re playing, if an advert comes on he comes to watch it, or copy it.” – IDI 6, mother of a 8 month old boy*

*“A lot, they watch cartoons and he loves Mr. Bean, they watch it and even imitate him, they… they watch a lot of TV, like even when the adverts come on they imitate all that’s being said and done. Yoh, they have a time when they’re done playing and they ask to watch TV, maybe like an hour, but sometimes they get bored when they figure out that it’s not what they like watching that’s on the TV playing, but an hour ja.” – IDI 7, mother of a 12 month old boy*

Often, children watched TV because their mother or the rest of the family was watching, or because the mother needed to do something else, like clean or cook, and used the TV as a babysitter. When asked whether mothers thought that TV was good or bad for children, the responses were again varied; but most mothers thought that TV was beneficial and served as a means for teaching children to talk. A few mothers said that TV made their children “lazy”, or that they were worried about access to social media on tablets or smartphones. These mothers restricted their children’s screen time.

*“Sometimes it depends because these kids fool us these days, you’ll think they’re doing something serious on the tablet but they’re on social media. But in terms of games, I think they’re good because you can see what the next step is. But hey, I don’t know. I won’t buy her a Tablet, I don’t even give her my phone.” – IDI 5, mother of a 7 month old girl*

*“I think it’s good because he learns stuff there are cartoons and cartoons have kids and they love them and they learn how to speak English from TV” - IDI 8, mother of a 13 month old boy*

## Discussion

This qualitative study aimed to examine mothers perceptions of play and physical activity in children up to the age of 2 years, and to understand any contextual barriers to enabling play that may exist in Soweto, South Africa. Our findings suggest that mothers perceived physical activity as important, but viewed it as an indicator of health rather than a behaviour that could be modified. While there was an understanding of the connection between physical activity and play with development, there was limited discussion about the promotion of play and child development. Many barriers to play were discussed, largely related to safety and poverty.

Interestingly, mothers in this study did not perceive physical activity as an independent behaviour, to be encouraged or promoted. However, mothers recognised that physical activity was an important sign that their infant was healthy; and that children who were overweight or underweight did not move as much as normal weight infants. Physical activity was thus a positive indicator of wellbeing. Indeed, it has been established that infants who are unwell, or undernourished are less physically active, and that activity improves with nourishment and improved health [9, 10]. Furthermore, physical activity in infancy has been associated with decreased adiposity, and with optimal BMI [11–13]. Therefore, these perceptions are intuitive and factually correct. However, there was a lack of understanding that physical activity as an independent behaviour was an important driver for growth and development. This is similar to findings from stakeholder focus group discussions conducted in South Africa following the release of the local movement guidelines for the early years, which found that parents had many gaps in their knowledge of the benefits of movement for young children [2, 4]. However findings from other countries – both high and low-income – have shown a better recognition of the importance of promoting physical activity in early childhood for health growth and development [14–16]. These perceptions imply that mothers in South Africa may not be motivated to encourage physical activity, and are not aware of the importance of physical activity for the healthy development of their infant.

Conversely, mothers in this study seemed to understand the importance of play for healthy development. In fact, developmental milestones appeared to be well known and frequently monitored by mothers, and appeared to carry more weight than other health outcomes. Therefore it is likely that mothers may be more motivated to change behaviour if improving developmental outcomes was the focus. This has been reported previously from mothers of older (preschool aged) children from South Africa [17] and is thus important to consider in intervention design. There also appeared to be misconceptions about the best means to promote development, whereby some mothers felt that expensive equipment was required for promoting child development. These are critical findings and often reflect the failure of early child development education to provide practical examples and means for mothers in low-resourced settings to become more health literate and easily apply the learning. Most play and physical activity seemed to occur naturally, during other day time activities such as bathing or feeding. However, there was some indication of mothers using structured play time for teaching moments, often imitating activities from creche. In South Africa, less than 50% of children under age three attend creche [18]. Therefore it is essential that opportunities for early learning occur in the home [8]. Most mothers did not read to their children, or even own a book. Similar findings were reported in the Stats SA General Household Survey in 2016, where only 20% of children in the lowest quintile of income were read to daily [18]. It seems that, while mothers do not lack the incentive or motivation to promote motor and cognitive development, they may lack the knowledge of how to do so.

Outdoor play is essential for childhood development, and should be encouraged to provide opportunities for learning, healthy growth, and increased physical activity [19]. However, there are major barriers to promoting play, especially outdoor play, within the Soweto context, and probably in many urban-poor neighbourhoods due to safety and financial constraints. While financial barriers prevented mothers from buying toys or paying for creche, some mothers were able to make adjustments such as making toys at home and ensuring learning was occurring within the home. However, lack of safety, fear of abduction, and structural barriers within their environment were difficult to overcome. A study conducted in Australia with mothers and fathers of 3–5 year old children also showed that safety concerns were a barrier to outdoor play, yet in that high-income setting the majority of fears were related to potential injury, and fewer fears about ‘stranger danger’ [14]. Systematic reviews citing studies from higher income settings such as Australia, UK, USA, and Canada have shown that concerns about child abduction, and ‘stranger danger’ are frequently reported as a deterrent to letting children play outside unsupervised [19]. However, the Position Statement states that “The odds of total stranger abduction are about 1 in 14 million based on RCMP [Royal Canadian Mounted Police] reports” [19]. In South Africa, according to crime statistics, the child abduction rate in 2016 was 1 in 100,000, being twice as high in females compared to males. Therefore, the risks in South Africa do not fall within the realm of normal or acceptable risks associated with outdoor play according to the Position Statement on Active Outdoor Play published in Canada [19], and may thus outweigh the benefits in crime ridden settings. Research conducted on young nulliparous women in Soweto showed that lack of safety, and lack of facilities, as well as financial constraints made it very difficult for them to be sufficiently physically active [20]. These barriers are potentially being transferred to the next generation, thus perpetuating a cycle of physical inactivity and resulting in increased obesity rates, non-communicable disease risk; and poorer developmental outcomes linked to economic decline [8].

There was a mixed view on the role that gender had in types of play and perceptions of physical activity amongst young children. Some mothers reported that each child was unique in their affinity for physical activity and that gender did not play a role. However, there were other mothers who had strong views about the role of gender in play. These views, while mixed in this sample, are important for understanding differing opportunities that may be provided for play and physical activity according to gender. In a sample of infants from Soweto, objectively measured physical activity was significantly higher in boys compared to girls from three months of age [11]. Furthermore, mothers of girls reported that their infants spent less time playing outside than mothers of boys [11]. Similarly, in a qualitative study conducted with rural mothers of 3–5 year olds in Bangladesh, most mothers reported that boys and girls do, and should, play differently, and that girls were often forbidden from going outside [15]. In higher income settings, natural outdoor play has been found to be more gender neutral, offering better gender equity [19], however cultural norms in these lower income settings seem to result in contrasting beliefs.

Young women from Soweto have reported that men are provided more opportunities for physical activity than women, both due to cultural norms and actual facilities available for men; and that safety is a major barrier to participation in physical activity for women [20]. Differences in physical activity levels are evident across the life course in South Africa, with men being more physically active than women [11, 21]. Perceptions about gender roles in physical activity in conjunction with safety concerns which may be heightened with female children - and consequently opportunities being provided to children according to gender - are thus potentially setting up trajectories of lower physical activity and poorer health and developmental outcomes in females. It was also reported by a few of the mothers in this study that certain toys were for girls and others for boys, and that these should not be mixed. This was again similar to findings from the study in Bangladesh [15]. There was particular concern in the current study if boys wanted to play with ‘girls’ toys, as this may result in homosexuality and was frowned upon culturally. It has been established that these sex role socialisations occur very early [22], and may provide another barrier for equal play opportunities amongst children. It is therefore important to target these perceptions and sex-typed play early when initiating interventions in this context, and to be cognisant of how child gender and gender labelling may affect behaviours. However, cultural norms around gender must also be respected, such as the ancestral beliefs about gender roles mentioned by participants in this study. In Turkey, cultural gender roles seem to impact perceived parenting responsibilities around play [16]; whereby mothers feel solely responsible for enforcing play. Thus gender bias may be impacting both assumed parenting styles, and their perceptions about how their children should play.

Lastly, access to screen time amongst these infants was problematic. Most mothers reported that screen time was freely available to infants at a very early age, and that they had the autonomy to choose whether or not to watch TV. Given that multiple family members often share one living space in households in Soweto, infants are likely to be in the same room in which the TV is switched on – and are thus unable to move away from the TV, but could choose to engage or not. In this age group, screen time is not recommended at all [1, 4]. However, previous studies across different settings have shown that screen time is high in this age group, and detrimentally associated with adiposity, motor development, and language development [11, 12, 23]. A few mothers thought that screen time was beneficial for infants, providing opportunities for learning – particularly language. Similarly, the majority of parents in an Australian study believed that screen time was beneficial for learning and for language development [14]. In fact, studies have found that TV time is associated with language delay in infants [24, 25], and that toddlers’ (aged two) ability to self-regulate media exposure was dependant on their emotional development [26]. According to international and national guidelines, screen time should be avoided as much as possible in this age group, and alternatives that provide stimulation and opportunity for learning should be incorporated. However, limiting screen time may be particularly difficult for mothers who are looking after children on their own and may have limited resources or time to find alternatives. Since it seems that some mothers in this sample believed that TV time is beneficial, this misconception needs to be addressed in order to optimise early childhood development, and possibly allow for more opportunities for play instead.

Key recommendations for intervention development to promote play in this setting include: 1) focussing intervention content on the promotion of child development (which resonated best with mothers) through movement, rather than on increasing physical activity or play; 2) providing mothers and caregivers with contextually relevant information on how to promote development through play, including information on how to promote achievement of milestones without the use of equipment or resources; 3) educating mothers on the recommended movement guidelines for the early years, with a particular focus on the detrimental effects of screen time in this age group and examples of stimulating alternatives; and 4) ensuring that parent and child gender is considered when developing intervention material, and that sex-typed play is avoided and gendered roles in play are discussed.

This study has limitations, including the risk that certain participants may bias the results by dominating the discussion. We minimised this risk by using trained facilitators who encouraged group discussion and participation. We also verified the themes that emerged from the FGDs by conducting the IDIs and ensuring that themes persisted into the IDIs before being considered in the thematic analysis. All qualitative analysis is susceptible to interpreter bias, however we attempted to minimise this bias by double-coding all transcripts, and ensuring that there was agreement amongst multiple researchers, with a fourth external researcher affirming the coding framework and thematic analysis. We were required to make assumptions in our interpretations of some themes. This study did not examine the perspectives of fathers or other caregivers, and future research should aim to understand whether these perspectives differ, and how they may be influencing behaviour and decision making. Based on that and the specific population sampled, the findings of this study may not be generalisable to other populations or contexts.

## Conclusions

In conclusion, this study has shown that while mothers are aware of their infants’ physical activity, they considered this as an indicator of health and wellbeing rather than a modifiable behaviour. Developmental attainment was an important outcome for mothers, and they were somewhat aware of how play could improve development, yet wanted more information. It would be important to determine whether providing this information to mothers results in changes in infants activity. There were many perceived barriers to promoting play and physical activity, including financial barriers, lack of safety, and social norms and cultural perceptions. Furthermore, screen time was not often restricted and was freely available to children. We believe that future studies which address these perceptions and barriers when designing interventions in this context will allow for equal opportunities for play to be provided to all children.

## Supplementary information

**Additional file 1.** Exploratory guide for focus group discussions.

**Additional file 2.** Exploratory guide for in-depth interviews.

## Data Availability

The datasets used and/or analysed during the current study are available from the corresponding author on reasonable request.

## Abbreviations

FGD: Focus group discussion
IDI: In-depth interview
ADHD: Attention deficit hyperactivity disorder
BMI: Body mass index
