# Supplementary material for: A qualitative study reporting maternal perceptions of the importance of play for healthy growth and development in the first two years of life
Source: BMC Pediatr. 2020 Sep 9;20:428. doi: 10.1186/s12887-020-02321-4 (PMC7487567; doi:10.1186/s12887-020-02321-4)
Supplement: Supplementary file 1 — Additional file 1. Exploratory guide for focus group discussions. [file 12887_2020_2321_MOESM1_ESM.docx]

**Infant Qualitative – Phase 1 – Exploratory Guide**

1. Definition
   1. What do you understand by the term wellbeing for babies?
   2. What words come to mind when we talk about the wellbeing of a baby?
2. Promotion
   1. What strategies do you use to promote the wellbeing of babies?
3. Personal
   1. Do you think your baby is well?
      1. Why?
   2. What do you do to ensure your baby is well?

| **Theme** | **Have they spoken about this?** | **If NO – ask this question** |
| --- | --- | --- |
| Growing/size/fat/thin |  | What do you understand by the words growth of a baby? |
|  |  | How do you think growth is related to wellbeing? |
| Health/infection /feeding |  | What do you understand by the words health of a baby? |
|  |  | How do you think health is related to wellbeing? |
| Development/activity/playing/learning/laughing/toys/crawling/sitting/movement/sleep |  | What do you think about the words development/movement of a baby? |
|  |  | How do you think development/movement is related to wellbeing? |
| Nurture/caregiving/looking after |  | What do you understand about the words caring for a baby? |
|  |  | How is caring related to wellbeing? |
