# Supplementary material for: A qualitative study reporting maternal perceptions of the importance of play for healthy growth and development in the first two years of life
Source: BMC Pediatr. 2020 Sep 9;20:428. doi: 10.1186/s12887-020-02321-4 (PMC7487567; doi:10.1186/s12887-020-02321-4)
Supplement: Supplementary file 2 — Additional file 2. Exploratory guide for in-depth interviews. [file 12887_2020_2321_MOESM2_ESM.docx]

1. ***General warm-up question about mother and infant health and development:***
2. When you think about the health of babies, what do you think you need to do for them to remain healthy? (Free listing, brainstorming. Interviewer listens and tries to see if women list physical activity. Write the answers in order they were given.)
   1. Probe: What are some of the woman’s worries related to the health of their babies?
   2. Probe: What are good ‘outcomes’ for a healthy child?
3. What do you understand by child development?
   1. What do you look at to see if your child is developing as they should for their age?
4. What do mothers in your community do to keep themselves healthy after pregnancy?
   1. What steps do you take in your life to remain healthy yourself?
   2. Probe: Why does this action/behavior keep you healthy?
   3. Probe: Who do you feel supports you to remain healthy (probe family, community, health worker)
5. ***Access to Information:***
6. What information do health workers and doctors/nurses give you about keeping your baby healthy?
   1. Where else do you get information or advice about keeping your baby healthy?
7. What information do health workers and doctors/nurses give you about your child’s development?
   1. Where else do you get information or advice about your baby’s development?
8. What information do health workers and doctors/nurses give you about playing with your baby?
   1. Where else do you get information or advice about playing with your baby?
9. Who is the most important provider of information about your baby’s health/wellbeing?

(Free listing, brainstorming. Facilitator/notetaker listens and tries to see if women list physical activity/play among the actions, and who is mentioned as a support. Write the answers in the order they are given.)

1. ***Caregiving:***
2. Could you please explain what typically happens after a mother gives birth to her baby? Who takes care of the newborn as soon as the baby is born?
   1. Probe: For how long is ____(name)____taking care of the newborn?
   2. Probe: What may prevent the mother from taking care of her newborn herself?
   3. Probe: What happens in the home or who helps the mother take care of the newborn?
   4. Probe: Do other caregivers looking after your baby help/limit your baby being active? How?
3. In your community, do the new mothers have to go back to work (field work, house work) soon after the baby is born? If so, how soon after the baby is born? How long is a mother away from her baby when she is at work? (Probe, using the following questions if they are not answered spontaneously.)
   1. Probe: In your community, do you see some issues/challenges when mothers need to go back to work? Who takes care of the baby?
   2. Probe: Do older siblings sometimes have to take care of younger children (babies, children <2yrs old), if yes, do you see any problems when children take care of younger children?
   3. Probe: Who feeds the baby when the mother is away and what is the baby fed?
4. In a women’s family, who helps babies to remain healthy and to grow and develop well?
   1. Probe: Which family members (mother-in-law, husband, sister-in-law) help and how do they help (talking, sharing the work, etc.)?
   2. Probe: Which family members prevent you/ limit you from making decisions about your child’s health and how do they do this?
   3. Who in your family has helped your children remain healthy and to grow and develop well?
   4. How have they contributed to your child’s health and development? What kinds of help have they offered?
5. In your **community**, who helps babies to remain healthy?
   1. Probe: What information do health care workers and doctors/nurses give about keeping your baby healthy and active?
   2. What information do health care workers and doctors/nurses give about promoting your child’s growth and development?
   3. Where or from who else do you get information about keeping your baby healthy and promoting his/her growth and development?
6. How do you maintain the bond between mother and baby?
   1. How is this bond affected when mothers need to go back to work?
   2. How important is this bond to you?

***3. Physical activity specific questions:***

Introduction/Explanation: Now we are going to talk about physical activity and playing, please feel free to give as many details as you can.

1. When you think of baby physical activity what comes to mind?
   1. Probe: Does playing count as being active?
   2. Probe: Do you believe that things such as floor play, tummy time, learning to crawl etc. count as being active?
   3. Probe: In the next few months what types of activities do you plan on promoting with your baby?
   4. Probe: In the next few years what types of activities do you plan on promoting with your baby?
2. In general, is your baby active?
   1. Probe: If no, is this a concern for you?
   2. Probe: Why is it important/not important for your baby to be active?
   3. Probe: What will happen if your baby is not active enough?
   4. Probe: Do you believe that having an active baby makes the baby more difficult to handle?
   5. Probe: What is active enough for a baby? What is too active?
   6. Probe: Do you believe that being too active is bad for your baby? Why?
3. What kind of things does your baby do when he/she is playing?
   1. Do you play with your baby? Who else plays with your baby?
   2. What types of toys does your baby play with?
   3. Do you think playing is important? Why?
4. What does your baby do on a normal day? (Tell me about a day in the life of your baby)
   1. Are there differences in the types of activities your baby should be doing according to age? List all activities across all developmental domains.
   2. Probe: What types of things do you do to keep your baby active?
   3. Probe: Are there differences in these activities according to babies’ age?
   4. Probe: Who should be helping your baby be active? How should they do this?
   5. Probe: Do you think active babies will become active children?
5. Are there differences in boy versus girl baby activities?
   1. Probe: Are boys ‘naturally’ more active than girls? Why?
   2. Probe: Should girls and boys be treated differently when playing? Should boys and girls be given different toys or access to facilities for playing?
   3. Probe: Would you worry if your boy was not active enough? Would you worry if your girls was not active enough?
6. In your opinion, what do you think are some of the major problems (barriers) women like you face when trying to ensure that their baby is active/plays enough?
   1. Probe for the following list of problems:

- Have to go back to work. Probe: What they do with their children when they go back to work?
- Feeling exhausted. Probe: Is there anyone who can help with the chores?
- Thinking activity is not healthy/necessary. Probe: Who tells them this?
- Being worried about the safety of their children. Probe: What are the concerns?

1. ***Home environment and neighborhood:***
2. Are there factors in your home environment that make it easier/ more difficult for your baby to be active/play and develop?
   1. Probe: Does your baby have access to play equipment/facilities/outdoor areas to play?
   2. Does your baby have access to toys, books etc.?
3. What kind of access does your baby have to playing equipment/space to play indoors and outdoors?
   1. Probe: If there is not much access, is this a problem for you?
   2. Probe: What type of play materials do you use most frequently?
4. Are there factors in your neighborhood that make it easier/ more difficult for your baby to be active?
   1. Probe: Are you worried about the safety of your baby when playing outside? What are you worried about?
   2. Probe: Are other children in your neighborhood playing outside? Why/why not?
   3. Probe: Are there any play spaces/places (e.g. park, soft play areas etc.) that you can take your child to play close to your home?
5. What types of activities do you do with your child to promote his/her development?
   1. Probe: Do you talk or sing to your child?
   2. Probe: Do you tell stories or read to your child?
   3. Probe: What types of games do you play with your child?

*(List all activities mentioned (e.g. toys, outings, reading, storytelling, singing, games etc.)*

d. Who does your child play with or who does these activities with him/her most of the time?

***5. TV/screen time:***

1. Do babies watch TV?
   1. Probe: Do you believe it is ok for your baby to watch TV/ use screens (such as tablets/cell phones etc)?
   2. Probe: How long is acceptable?
   3. Probe: Does age matter?
   4. Probe: Are there benefits to watching TV/using a cellphone or tablet for your baby?
2. When do you allow your baby to watch TV/ use a screen?
   1. Probe: Do you use the TV as a substitute for playing with/ entertaining your baby?
   2. Probe: Does your baby watch TV when you/other family members are watching TV?
   3. Probe: Are there other things you could be doing with your baby instead of watching TV?
3. Do you think it would be easy to limit the amount of time your baby spends in front of the TV?
   1. Probe: What are the barriers/facilitators?
   2. Probe: How much could TV time be feasibly reduced?
   3. Probe: Who in the family/home would make it difficult to limit TV time for your baby?

***6. Intervention feasibility:***

1. Do you think women in your community need help/support to keep their babies active, promote their development and provide them with opportunities to play?
   1. Probe: What kind of support would be useful?
   2. Probe: Do you think it will be possible to change these behaviours?
   3. Probe: Who would like to receive this kind of support?
   4. How would you like to receive the support and from whom?
2. Do you think group sessions/lessons with other women like yourselves would help?
   1. Probe: Who should deliver these sessions?
   2. Probe: What kind of information would be helpful in these sessions?
   3. Probe: Would you attend these sessions? Why/Why not?
   4. Probe: When and where should these be held? How often?
3. Are there any other ways that women can receive help/support/advice regarding their child’s growth and development?
